# Progress in Surgery

**Published:** 1898-02-19

**Authors:** 


### Progress in Surgery.

### GENITO-URINARY SURGERY.

**Urinary Calculi.**—Professor Ebstein (Gottingen) indicates the constant relation of an albuminoid substance to the formation of calculi, not only the formation of the nuclei, but also of the concentric accretions. Stones may be experimentally produced in animals by feeding them on oxamide.[1]

The diagnosis of renal calculus is often a matter of difficulty. Dr. William White[2] quotes two cases of calculus undetected even at the operations, and yet the stones were found to be present post-mortem. The Röntgen rays are likely to prove of great value for diagnosis in certain cases. Mr. Henry Morris[3] finds that calcium oxalate calculi throw the deepest shadows, and that in calculi the degree of opacity is mainly due to the calcium salts. Successful cases have been published. Thus Dr. James Swain[4] describes a case of a small oxalate calculus in the left kidney detected by skiagraphy. He confirms, by experiment, the conclusions of Henry Morris that calcium oxalate calculi are the most opaque to X-rays, their great density corresponding to their comparative impermeability. The diagnosis of stone in the kidney has been surveyed by George Heaton with a report of eight cases.[5] Certain diagnosis may only be possible by exploration of the kidney, which should not be delayed if the indications of stone be strong.

**Kidneys and Ureters.**—Dr. Tuffier (Paris) relates an experience of 156 operations on the kidney, and concludes that in traumatic lesions of the kidney conservative treatment is best; but that in tuberculosis early nephrectomy is indicated.[6] Dr. Gersuny (Vienna) refers to a case in which he was betrayed into error by a false result of catheterisation of the ureters. The kidney gave healthy urine, but proved after death to have been largely transformed into a purulent collection. Of 263 nephrectomies for malignant disease by Professor Küster (Marburg), only nine cases were without recurrence after three years; thus exemplifying the very great mortality.[6] Professor Jonnesco performs nephropexy by temporary sutures of silver wire, which pass through the skin, and are removed on the tenth day.

With regard to the surgery of the ureters it is shown experimentally that in anastomosis of the ureters it is well to preserve as far as possible the periureteral tissues and peritoneum intact.[7] J. W. Bovée publishes a successful case of reunion of divided ureters.[8] Ureterectomy has been performed for (*a*) tuberculosis, (*b*) suppuration in a dilated ureter, and (*c*) for hydro-ureter. Bazy[9] successfully removed a portion of ureter, and stitched the distal end to the bottom of the sac of a hydronephrotic kidney. The union took place over a catheter, which served as a drain for the urine, one end being brought out of the loin. In a second case the patient succumbed.

**Bladder.**—The absorptive power of the bladder for different solutions has been investigated by Morrow and Galbenbein.[10] Glucose, sodium chloride, urea, quinine, and carbolic acid were all freely absorbed, but morphine showed practically no absorption.

An analysis of over 200 cases of foreign body in the bladder is given by Dr. Packard (Philadelphia).[11] Of these 108 were surgical instruments, such as catheters or parts thereof, and in 17 of the cases medical men met with the misfortune. The remainder comprised domestic articles, *e.g.*, a toothpick, a cylinder of pork, a cork, a threepenny piece, and a vulcanised rubber watch-chain bar. In one case Reginald Harrison removed a calculus having for its nucleus the mouth-piece of a pipe, which the man persisted in asserting that he had swallowed a long time before. A case is also given of a piece of rubber catheter, which remained in the bladder for 17 years. The cystoscope is here of great service.

[1] Medical Week, Sept. 17, 1897. [2] Annals of Surgery, Jan., 1897. [3] Lancet, Nov. 14, 1896. [4] Bistol Medico-Chirurgical Journ., March, 1897. [5] Birmingham Med. Review, Sept., 1897. [6] Medical Week, Sept. 17, 1897. [7] Annals of Surgery, March, 1897. [8] Practitioner, March, 1897. [9] Revue de Chirurgie, May 10, 1897. [10] Zeitsch f. kl. Med., Vol. XXXIII., Nos. 1 and 2. [11] Annals of Surgery, May, 1897.

